# Contradiction in Star-Allele Nomenclature of Pharmacogenes between Common Haplotypes and Rare Variants

**DOI:** 10.3390/genes15040521

**Published:** 2024-04-22

**Authors:** Se Hwan Ahn, Yoomi Park, Ju Han Kim

**Affiliations:** 1 Department of Biomedical Sciences, Seoul National University Biomedical Informatics (SNUBI), Seoul National University College of Medicine, Seoul 03080, Republic of Korea; sehwanahn@snu.ac.kr; 2 Seoul National University Biomedical Informatics (SNUBI), Seoul National University College of Medicine, Seoul 03080, Republic of Korea; yoomip@snu.ac.kr; 3 Medical Research Center, Seoul National University College of Medicine, Seoul 03080, Republic of Korea

**Keywords:** pharmacogenomics, haplotype, star allele, haplogroup, rare variant, 1000 Genomes Project

## Abstract

The nomenclature of star alleles has been widely used in pharmacogenomics to enhance treatment outcomes, predict drug response variability, and reduce adverse reactions. However, the discovery of numerous rare functional variants through genome sequencing introduces complexities into the star-allele system. This study aimed to assess the nature and impact of the rapid discovery of numerous rare functional variants in the traditional haplotype-based star-allele system. We developed a new method to construct haplogroups, representing a common ancestry structure, by iteratively excluding rare and functional variants of the 25 representative pharmacogenes using the 2504 genomes from the 1000 Genomes Project. In total, 192 haplogroups and 288 star alleles were identified, with an average of 7.68 ± 4.2 cross-ethnic haplogroups per gene. Most of the haplogroups (70.8%, 136/192) were highly aligned with their corresponding classical star alleles (VI = 1.86 ± 0.78), exhibiting higher genetic diversity than the star alleles. Approximately 41.3% (N = 119) of the star alleles in the 2504 genomes did not belong to any of the haplogroups, and most of them (91.3%, 105/116) were determined by a single variant according to the allele-definition table provided by CPIC. These functional single variants had low allele frequency (MAF < 1%), high evolutionary conservation, and variant deleteriousness, which suggests significant negative selection. It is suggested that the traditional haplotype-based naming system for pharmacogenetic star alleles now needs to be adjusted by balancing both traditional haplotyping and newly emerging variant-sequencing approaches to reduce naming complexity.

## 1. Introduction

Pharmacogenomics (PGx) is the study of how an individual’s genetic makeup affects their response to medications, with a focus on understanding genetic variations in drug transporters, receptors, and metabolic enzymes [1,2]. This knowledge has the potential to greatly improve medication efficacy and safety, as well as reduce the risk of adverse drug reactions [3]. The advent of next-generation sequencing (NGS) technologies has significantly advanced PGx by facilitating the discovery of rare functional genetic variants [4,5]. In response to these technological advancements, the star-allele nomenclature has become a critical component in PGx [6]. Its primary purpose is to establish a standardized, widely recognized system for classifying genetic variations, ensuring clear and precise communication within the scientific and medical communities [7]. The star-allele system not only provides names for genetic variations, but also plays a crucial role in predicting the functional effects of genetic differences in pharmacogenes. Traditionally, this is achieved by representing combinations of single-nucleotide polymorphisms (SNPs) and/or small insertions and deletions (INDELs), known as haplotypes, that can influence protein function [8]. A haplotype refers to a set of genetic variants located on a single chromosome. Various professional societies, such as the Clinical Pharmacogenetic Implementation Consortium (CPIC) [9], the Dutch Pharmacogenetics Working Group (DPWG) [10], and the American College of Medical Genetics and Genomics (ACMG) [11], provide clinical PGx guidelines to optimize therapy for individual patients [12].

Traditionally, it should be noted that the purpose of the star-allele nomenclature is to define haplotypes, but in practical application, many defined star alleles represent individual rare functional variants instead of combinations of variants. As of 21 October 2021, the majority of star alleles defined in CPIC guidelines, approximately 85.6% (716 out of 836), are defined by a single rare functional variant in the definition table (Figure S1). For instance, the CYP2D6*3 allele, classified as a no-function allele, is characterized by a single nucleotide deletion, rs35742686 in the CPIC definition table. This deletion results in a frameshift, leading to premature truncation of the CYP2D6 protein and a loss of enzyme function [13]. The rapid discovery of rare functional PGx variants has led to excessive complexity in the traditional haplotype-based star-allele nomenclature. This complexity in pharmacogenomic knowledge and its nomenclature is believed to be a barrier to the clinical application of PGx by prescribing clinicians, potentially delaying its adoption in both clinical and research settings.

In this study, we aim to evaluate the impact of the rapid introduction of many rare functional variants on classical star-allele nomenclature, which is traditionally haplotype-based (Figure S2). To accomplish this, we developed a novel method that iteratively eliminates rare and functional variants to construct haplogroups that represent the common ancestry structures. Lastly, we analyzed the genomic properties [14,15] of these rare functional-based star alleles using six genomic features: the number of variants determining star alleles, allele frequency, GERP++ conservation score, and in silico deleteriousness scores including SIFT, Poly-Phen-2, and CADD. This study not only offers a novel perspective on pharmacogene classification but also contributes to the broader understanding of genetic diversity and its implications in pharmacogenomics.

## 2. Materials and Methods

### 2.1. The 1000 Genomes Project

The 1000 Genomes Project (1KGP) is a comprehensive resource that provides a representation of human genetic variation through the sequencing of 2504 individuals from 26 countries, divided into five main population groups: Africa (AFR), America (AMR), Europe (EUR), East Asia (EAS), and South Asia (SAS) [16]. It offers valuable information for evolutionary, functional, and pharmacogenomic studies of human genetics. For our study, we downloaded the variant call format (VCF) files of 2504 individuals from the 1000 Genomes Project phase III dataset [17].

### 2.2. Functional Variant Determination

It is essential to determine functional variants before constructing haplogroups. In this study, we initially selected 25 pharmacogenes classified as CPIC Level A or A/B, based on gene regions according to the Ensembl of GRCh37 human assembly [18]. Functional variants were defined for each pharmacogene using the Ensembl Variant Effect Predictor (VEP, version 104.3) tool [19]. A variant was determined functional if it met either of the following criteria: (1) The ”Impact” field in the VEP annotation was labeled “HIGH” or ”MODERATE”, indicating a potential effect on the gene or its product’s structure and function. (2) The Combined Annotation Dependent Depletion (CADD) score [20] of the variant was above 15, ranking it within the top 5% of deleterious variants in the human genome. Additionally, variants with a minor allele frequency (MAF) of less than 1% in the 1KGP were considered rare variants.

### 2.3. Constructing Haplogroups

**Figure 1:**
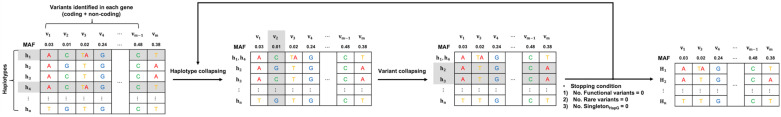
Process for constructing haplogroups for each pharmacogene. Initially, a matrix is created, with each row representing a phased allele sequence (haplotype, h_n_) and each column representing all observed variants, including coding and non-coding variants, within a gene. During the haplotype collapsing step, all identical haplotype sequences are combined into a single entity. Then, the variant with the lowest minor allele frequency (MAF) is removed from the matrix (variant collapsing). These two steps are repeated until the stopping condition. The stopping condition is the MAF.

We generated a matrix containing all observed variants, including single-nucleotide variants (SNVs) and insertions/deletions (INDELs), along with their corresponding haplotypes, from the phased data of the 1KGP for each of the 25 pharmacogenes (Figure 1). The process of haplotype collapsing was then applied to merge identical sequences that are haplotypes within the matrix into a single entry. Subsequently, in a step referred to as variant collapsing, the variant with the lowest minor allele frequency (MAF) was removed. These two steps were iteratively repeated until the MAF met the stopping condition. The stopping condition was defined as the complete absence of rare and functional variants and the absence of singleton_HapG_, which consists of a single haplotype. Finally, a haplogroup was defined as a collection of haplotypes with identical genetic variations that were free from both rare and functional variants, with each row in the matrix representing a distinct haplogroup.

### 2.4. Assignment of Star Alleles

In pharmacogenomics, haplotypes, which are combinations of inherited variants such as single-nucleotide polymorphisms (SNPs), insertions/deletions (INDELs), and structural variants (SVs), are identified as star alleles (*). Our focus was on 25 pharmacogenes that are classified with evidence levels A or A/B according to the Clinical Pharmacogenetics Implementation Consortium (CPIC) based on data from the 1000 Genomes Project (1KGP). The 25 pharmacogenes are as follows: *IFNL3*, *GSTP1*, *CYP2D6*, *VKORC1*, *NUDT15*, *NAT2*, *UGT1A1*, *G6PD*, *CYP4F2*, *GSTM1*, *UGT2B15*, *TPMT*, *CYP2B6*, *CYP3A4*, *CYP3A5*, *CYP2C8*, *CYP2C9*, *NAT1*, *UGT1A4*, *CACNA1S*, *SLCO1B1*, *RYR1*, *CYP2C19*, *CFTR*, and *DPYD* (Table S1). To assign star alleles to individuals from the phased VCF file of the 1KGP, we utilized PyPGx v0.20.0 [21] with the Human Genome version (hg19).

### 2.5. Evaluation

To evaluate the constructed haplogroups, we utilized the variation of information (VI) index, a metric based on principles of information theory and entropy [22]. The VI index quantifies the information loss and gain during the transition from one clustering to another, enabling us to assess the similarity between established haplogroups and star alleles for each pharmacogene. The VI index values range from 0, indicating a perfect match in clustering, to $\log_{2}N$ , denoting completely distinct clusterings, where N is the total count of haplotypes, which in the case of the 1KGP is 5008. The VI index values were computed using the “mclust” package in R [23].

Additionally, we computed Nei’s standard genetic distance [24] to assess genetic diversity among five populations. This metric reflects the degree of genetic divergence or differentiation between compared populations, with higher values indicating greater divergence. This calculation was applied to evaluate the genetic distance within each frequency of star alleles and haplogroups.

### 2.6. Enrichment Analysis

We conducted enrichment analysis using the hypergeometric test to identify the associations between newly constructed haplogroups and pre-existing star alleles. This process was important for identifying whether specific star alleles were more frequently found within certain haplogroups than would be expected by chance.

In this analysis, we treated each star allele as a distinct category and compared the observed frequency of each allele within haplogroups to its expected frequency, which was calculated based on its overall distribution among all haplogroups. Then, significant associations between haplogroups and star alleles were identified if the False Discovery Rate (FDR) was less than 0.05.

Following this, we classified all the star alleles identified in the 1KGP into two groups based on their association with haplogroups. The first group, defined as S_A_, included star alleles that showed a statistically significant association with at least one haplogroup. Conversely, the second category, named S_I_, comprised star alleles that did not exhibit any significant association with haplogroups and were thus considered to be independent of haplogroups. Next, we conducted a comparative analysis to highlight the differences between the star alleles associated with haplogroups (S_A_) and those independent of haplogroups (S_I_).

### 2.7. Genomic Features of Star Alleles

In order to investigate the star alleles defining variants that are not tied to specific haplogroups, we employed in silico pathogenic prediction scores to gauge their evolutionary conservation and potential deleterious effects. Variants with a GERP++ score higher than 2 were regarded as being evolutionarily conserved and potentially functional [25]. Furthermore, we utilized the SIFT score, categorizing variants with scores below 0.05 as deleterious, suggesting a likely deleterious impact on protein function [26]. Additionally, we assessed these variants using PolyPhen-2 (PP2), where scores exceeding 0.5 were indicative of potential deleterious effects on protein structure or function [27]. Additionally, we utilized the CADD score, with a cutoff value set above 15 [20]. By employing this comprehensive approach, we were able to thoroughly analyze the genetic variants relevant to haplogroup-independent star alleles, offering insights into their evolutionary history and potential pathogenicity.

## 3. Results

In our study, we adhered to CPIC guidelines, which are widely accepted as the standard for star-allele nomenclature [9]. The CPIC definition tables show that the star-allele nomenclature is mixed with both traditional haplotype-based and rare functional-variant-based approaches. To navigate the intricacies of these interwoven nomenclatures, we introduced the concept of haplogroups, which represent common ancestry structures. We hypothesized that traditional haplotype-based star alleles are closely linked to haplogroups. Therefore, we investigated the impact of rare functional variants on the established star-allele system through the construction and analysis of haplogroups.

### 3.1. Haplogroup Construction

We constructed haplogroups for 25 pharmacogenes, utilizing all variants found in the 1KGP, which includes both coding and non-coding variants. The functional impact of these variants was determined using the Ensembl Variant Effect Predictor (VEP) tool, based on the GRCh37 human genome assembly. The criteria for identifying rare and functional variants were detailed in the methods section.

The process of constructing haplogroups for each pharmacogene began with the creation of a matrix containing all variants found in 2504 genomes, including INDELs and SNVs (Figure 1). Our approach involved two main steps: haplotype collapsing and variant collapsing. Two main steps were involved in the approach: haplotype collapsing and variant collapsing. In the haplotype collapsing step, all identical sequences in the matrix were combined into a single entry. In the variant collapsing step, the variant with the lowest minor allele frequency (MAF) was removed. These steps were repeated iteratively until all functional and rare variants were excluded and no singleton_HapG_ remained, which is a haplogroup defined as consisting of a single haplotype. Finally, haplogroups were constructed for every 25 pharmacogenes using the 1KGP.

Our analysis yielded an average of 7.68 ± 4.2 haplogroups per pharmacogene, with each haplogroup comprising 2.1 ± 1.1 genetic variations (Table 1). In terms of star-allele nomenclature, we observed that approximately 76.4% (8.8 out of 288) of the star alleles were also defined as single variants within the 1KGP. This suggests rare functional-variant-based nomenclature interwoven with traditional haplotype-based nomenclature.

**Figure 2:**
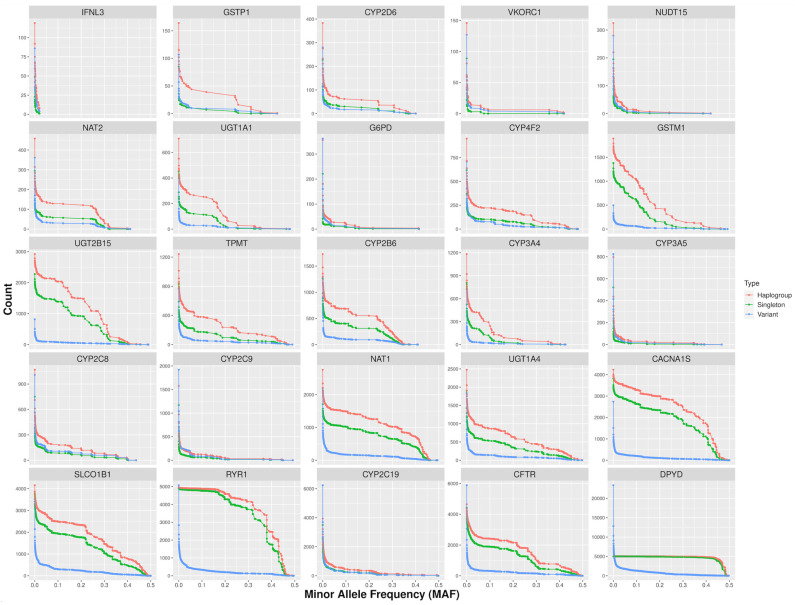
The number of haplogroups, singletons, and variants across iterations until the minor allele frequency (MAF) satisfies the stopping condition.

In the process of establishing haplogroups for various pharmacogenes, the criteria for the stopping condition differed across genes (Figure 2). Our analysis across 25 pharmacogenes showed an average MAF of 0.4 at this stopping condition. For instance, the gene *DPYD* had the highest MAF at the stopping condition (0.497), while *IFNL3* was the lowest (0.02). Additionally, we observed that *DPYD* was the longest gene in our study, while *IFNL3* was the shortest. It led to a positive correlation between the length of a gene and its MAF at the stopping condition, as demonstrated by a Spearman correlation coefficient of 0.8 (*p* < 0.01, Figure S3). This relationship suggests that longer genes, such as *DPYD*, tend to accumulate a broader range of variants, including those with higher MAFs, due to their greater potential for evolutionary adaptability and genetic diversity [28,29]. In contrast, shorter genes, such as *IFNL3*, have fewer variants and reach the stopping condition with lower MAFs, indicating a reduced capacity for genetic and evolutionary change.

### 3.2. Evaluate Haplogroup Construction

**Figure 3:**
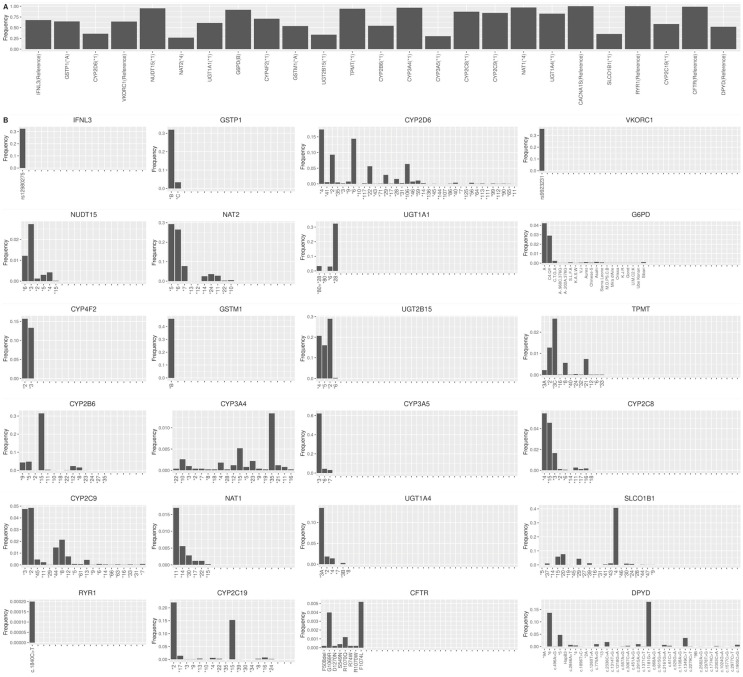
Distribution of star alleles in the 1KGP. The frequency of star alleles observed within the 1KGP dataset of 25 pharmacogenes was assigned using PyPGx (version 0.20.0). (**A**) Allele frequencies of reference star alleles of each gene are shown. (**B**) Allele frequencies of non-reference star alleles are shown.

To evaluate the effectiveness of our haplogroup construction method, we used the star alleles observed in the 1000 Genomes Project (1KGP) to analyze both similarity to the constructed haplogroups and population diversity. Using PyPGx v0.20.0 and based on Human Genome version 19 (hg19), we assigned star alleles for 25 pharmacogenes with CPIC evidence levels A or A/B to each individual in the 1KGP. Our analysis revealed a distinct distribution of star alleles across these pharmacogenes, with an average frequency of 0.03 for non-reference star alleles and a higher average of 0.70 for reference star alleles (Figure 3). Notably, for genes like *CACNA1S*, all identified star alleles were classified as the reference allele, whereas *RYR1* predominantly featured the reference star allele, with a rare exception of a unique haplotype defined by genetic variant c.1840C>T. Furthermore, genes such as *DPYD* and *CYP2D6* displayed considerable allele diversity, with 39 and 38 different star alleles identified, respectively.

To quantify the similarity between our constructed haplogroups and the star alleles, we utilized the variation of information (VI) index, a measure based on information theory principles. We categorized the VI index values equally into four groups representing different levels of association: strong, moderate, weak, and no association. A stronger association implies a higher degree of similarity between the haplogroups and star alleles. Our analysis revealed that, except for *GSTM1* and *DPYD*, the genes demonstrated a strong association between their haplogroups and star alleles, as indicated by the VI index evaluations (Table 1). These two genes showed a moderate association. Notably, none of the genes fell into the categories of weak or no association. This outcome validated the effectiveness of our haplogroup construction, confirming that these haplogroups accurately represent the star alleles and exhibit a strong correlation between them. Moreover, we observed that shorter genes displayed a more robust association with their haplogroups than longer genes, as evidenced by a Spearman correlation coefficient of 0.57 (*p* < 0.01, Figure S3).

**Figure 4:**
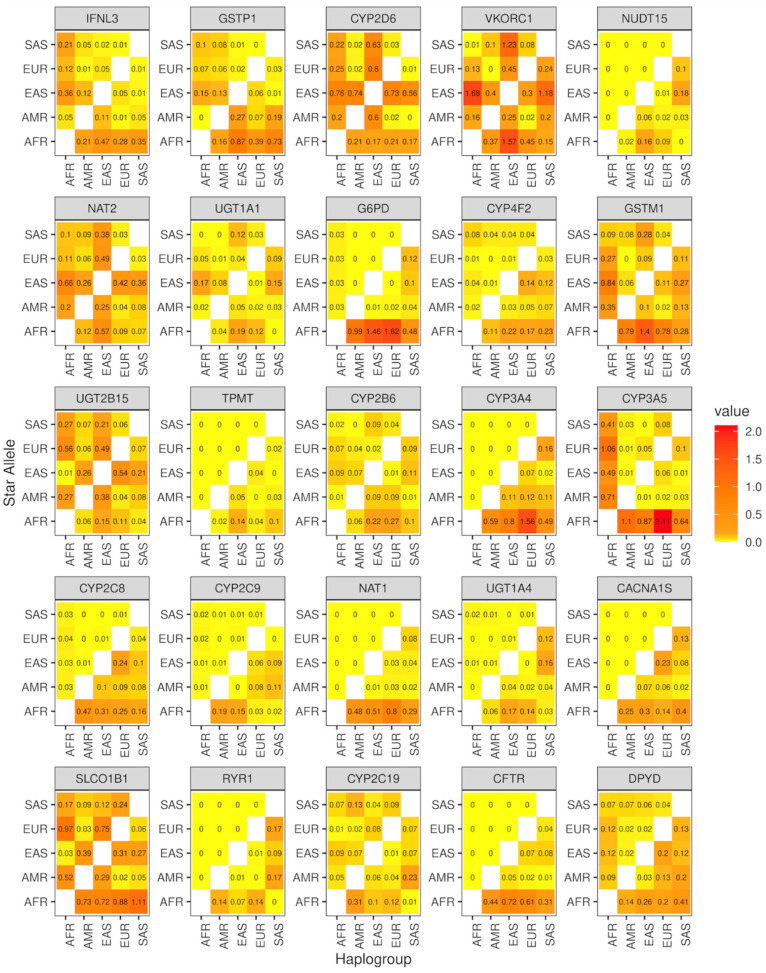
The heatmaps of Nei’s standard genetic distance for 25 pharmacogenes across five populations. In each panel, the upper triangular part of the matrix shows the results from star alleles, while the lower triangular part shows the results from haplogroups. AFR, African; AMR, American; EUR, European; EAS, East Asian; SAS, South Asian.

Furthermore, we explored the potential of haplogroups and star alleles in differentiating global populations using Nei’s standard genetic distance (Figure 4). Specifically, we computed the genetic distance between five major global populations, Africa (AFR), America (AMR), Europe (EUR), East Asia (EAS), and South Asia (SAS), for both star alleles and haplogroups. Our results showed that haplogroups were better at reflecting genetic diversity compared to star alleles (*p* < 0.01, Wilcoxon test).

### 3.3. Genomic Characterization of Star Alleles by Haplogroups

**Figure 5:**
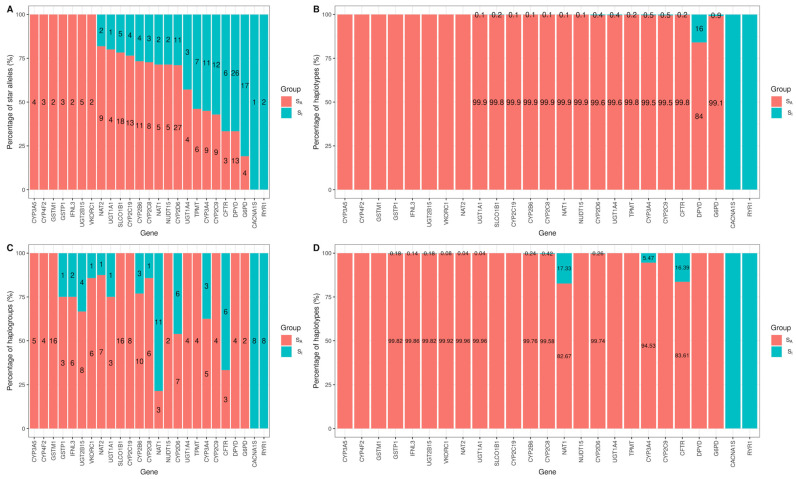
The percentage of star alleles and haplogroups that have a significant association in the 1KGP. (**A**) The percentage of star alleles that have a significant association with at least one haplogroup in the 1KGP. (**B**) The percentage of haplotypes representing respective star alleles. (**C**) The percentage of haplogroups that have a significant association with at least one star allele. (**D**) The proportion of haplotypes respective haplogroups. Only the values under 100% are represented. S_A_, star alleles that have a significant association with haplogroups; S_I_, star alleles that are independent of haplogroups.

In our study, we aimed to explore the associations between traditional haplotype-based star alleles and their ancestral haplogroups by conducting an enrichment analysis. We focused on the star alleles identified in the 1KGP and categorized them into two groups based on the enrichment test: those associated with haplogroups (S_A_) and those not (S_I_). Our findings revealed that 58.7% (N = 169/288) of the star alleles were classified as S_A_, indicating a strong association with ancestral lineages (Figure 5A). Remarkably, for genes such as *CYP3A5*, *CYP4F2*, *GSTM1*, *GSTP1*, *IFNL3*, *UGT2B15*, and *VKORC1*, all star alleles exhibited significant associations with haplogroups. Additionally, over 91.7% of the haplotypes within these genes were categorized as S_A_, indicating that this category encompasses the majority of haplotypes in the 1KGP (Figure 5B). The remaining 8.3% of haplotypes not classified as the S_A_ category were primarily due to the gene *DPYD*, which had the highest number of haplotypes assigned to the S_I_ category (Figure S28). For genes *CACNA1S* and *RYR1*, the enrichment test was not feasible as only reference star alleles were present, with no association with the S_A_ category detected (Figures S23 and S25). Furthermore, when analyzing from the haplogroup perspective, about 70.8% (N = 136) of haplogroups were closely linked to S_A_ star alleles (Figure 5C). Moreover, 90.3% of haplotypes belonged to these S_A_ category (Figure 5D). Similar to the star alleles, *CACNA1S* and *RYR1* showed no significant haplogroup associations. The associations between haplogroups and traditional haplotype-based star alleles were represented for 25 pharmacogenes (Figures S4–S28).

**Figure 6:**
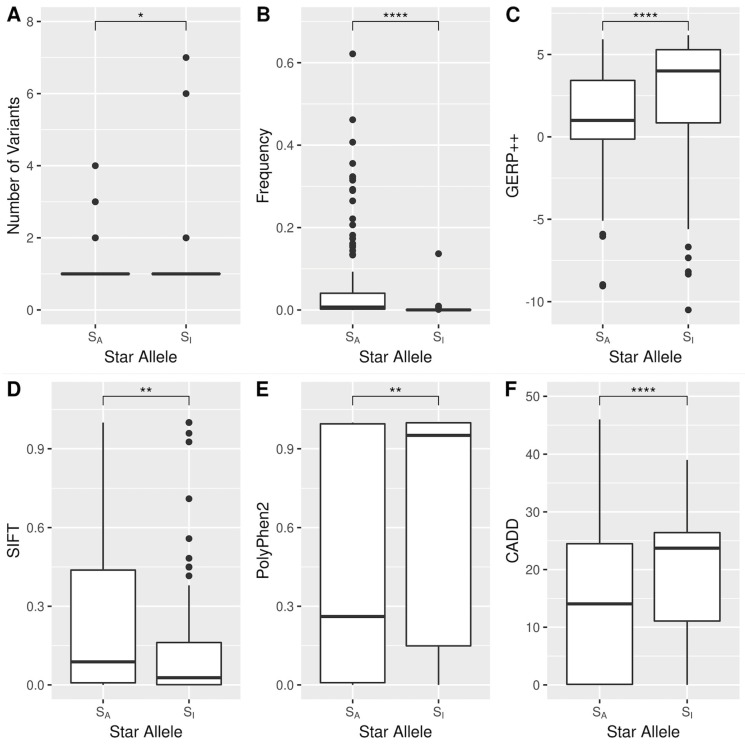
Genomic characterization of star alleles that are significantly associated with haplogroups (S_A_) and star alleles that are independent of haplogroups (S_I_) with the six genetic molecular features. (**A**) The number of variants defining star alleles. (**B**) The frequency of star alleles in the 1KGP. (**C**–**F**) GERP++, SIFT, PolyPhen2, and CADD score. * *p* < 0.05; ** *p* < 0.01; **** *p* < 0.0001 by the Wilcoxon test.

We conducted a further investigation of the genomic properties of S_A_ and S_I_ star alleles. Our findings show a statistically significant difference in the number of variants between these groups. On average, S_A_ alleles contained 1.49 variants, while S_I_ alleles had slightly more, averaging 1.67 variants per allele (Figure 6A). Notably, about 76.7% (104 out of 136) of the S_I_ alleles were characterized by a single variant (Figure S29). S_I_ alleles were also much rarer than S_A_ alleles, typically appearing at frequencies below 1% (Figure 6B). Furthermore, S_I_ alleles demonstrated higher evolutionary conservation, as evidenced by their significantly higher GERP++ scores (Figure 6C). This conservation suggests stronger selective pressures over evolutionary time. Additionally, differences in deleteriousness scores, such as SIFT, CADD, and PolyPhen-2 (PP2), were significant, with S_I_ alleles showing more deleterious effects, indicating that these variants are likely to have a greater impact on protein function (Figure 6D–F).

Considering the low frequency and high evolutionary conservation of S_I_ star alleles, along with their potential deleterious impacts on protein function, it is likely that these alleles are influenced by negative selection. This contrasts with the typical pharmacogenetic variants, which have not been significantly shaped by evolutionary pressures due to the recent nature of drug exposure in human history [30]. Therefore, it is suggested that the traditional haplotype-based pharmacogenetic star-allele nomenclature is now required to balance the traditional haplotype-based and the newly emerging functional-variant-based approaches to accelerate clinical adoption.

## 4. Discussion

The star-allele nomenclature is continually updated by various consortiums such as CPIC, PharmGKB [31], and PharmVar [32] to incorporate new haplotypes that affect drug metabolism. However, the traditional system for naming star alleles, which relies on haplotypes, has become substantially more complex due to the rapid discovery of rare functional variants in pharmacogenomics, accelerated by advancements in next-generation sequencing (NGS) technology. This complexity of the system can make it difficult for users to interpret and apply genomic data accurately. Recognizing these challenges, our study explored the traditional haplotype-based nomenclature.

We aimed to evaluate how the influx of numerous rare functional variants influences the traditional haplotype-based nomenclature used for identifying star alleles. To address this, we proposed a novel approach that involves the construction of haplogroups, representing common ancestry, by systematically excluding both rare and functional variants from the 1KGP of 25 pharmacogenes. This analysis identified an average of 7.68 haplogroups, which exhibited a high similarity to the existing star alleles based on the variation of information. Moreover, the haplogroup displayed greater genetic diversity than the corresponding star allele, which can be attributed to their common ancestor.

Our investigation of star alleles through haplogroup analysis shows the complex connection between traditional haplotype-based alleles and their shared ancestral haplogroups. By performing enrichment analysis, we discovered significant relationships between star alleles and haplogroups across pharmacogenes. Our study found that over half of the alleles were connected to their ancestral haplogroups, with 66.6% being classified as S_A_ star alleles. This indicates a strong association between many star alleles and their haplogroups, highlighting the importance of considering ancestral lineage in pharmacogenomic studies.

In examining S_A_ and S_I_ star alleles, we observed distinct genetic characteristics. S_I_ alleles, which are less frequent and show higher evolutionary conservation, may significantly impact protein function and appear to be influenced by negative selection. In contrast, S_A_ alleles are more common and exhibit lower evolutionary conservation. Although the current nomenclature integrates both S_A_ and S_I_ star alleles, our findings highlight significant differences between them in terms of frequency and evolutionary conservation. Consequently, we suggest an adjustment in the pharmacogene nomenclature to treat these types of alleles separately. This modification would enhance the precision of genetic interpretations and facilitate their clinical application by providing clearer, more actionable genetic information.

The differentiation of star alleles, particularly those classified as S_I_ defined by the impact of a single variant, suggests a potential pathway to adjust the current star-allele nomenclature. By more clearly categorizing these alleles, we hypothesize that the complexity of the naming conventions could be reduced, which might simplify genetic data interpretation for clinical practitioners. Such adjustments would aim to support clearer communication and practical application of pharmacogenetic information in clinical settings. However, further empirical studies are needed to validate whether these changes indeed lower barriers to clinical adoption and expedite the integration of personalized medicine into practice.

Our method for constructing haplogroups of pharmacogenes has its limitations. It involves collapsing steps, which exclude certain variants. All variants found in a specific gene will be removed if the MAF of the functional variant to be removed is the highest among them. In cases where no suitable variants are left for haplogroup formation, we cannot define them, resulting in all alleles being categorized as singleton_HapG_. Furthermore, our study has several limitations in addressing broader genetic factors that influence drug response. First, we did not consider copy number variants (CNVs), which can significantly affect gene expression and enzyme levels, thereby impacting drug metabolism. Second, we did not investigate the inheritance patterns of variants—whether they are inherited on the same chromosome (in cis) or on different chromosomes (in trans)—which is vital for accurately understanding how these variations function together and affect clinical outcomes. Third, we omitted analysis of the co-inheritance of variants across different pharmacogenes, a key factor in predicting multi-drug interactions and adverse drug reactions. Recognizing these limitations, future research should aim to include these aspects to provide a more comprehensive understanding of pharmacogenomic variability and enhance the predictive accuracy of drug response models.

Our study utilizes the CPIC guidelines available up to October 2021. It is important to note that any updates to these guidelines following this date represent a potential limitation, as they may introduce changes not reflected in our analysis. These updates could affect the long-term relevance and applicability of our findings. Additionally, while our study relies on CPIC for genetic variation information, PharmVar is another valuable resource that could be leveraged for such data. This alternative database offers a comprehensive catalog of genetic variations that may provide additional insights, suggesting a broader approach to data selection could enhance the robustness of future research in pharmacogenomics.

Our study utilized Combined Annotation Dependent Depletion (CADD) scores to identify functional variants, which assess both coding and non-coding variants. However, incorporating additional computational tools, such as FathmmXF [33], could enhance our methodology by offering alternative predictions on the impact of genetic variations. Integrating these tools would refine our approach to defining and understanding haplogroups in pharmacogenomics.

In conclusion, our study introduced a method for constructing haplogroups that reflect common ancestry for pharmacogenes and evaluated the impact of the numerous rare functional variants in traditional haplotype-based star-allele nomenclature. Our findings emphasize the need for an advanced nomenclature that incorporates these insights, enabling clinicians to better predict patient responses to medications and tailor treatments accordingly. As we continue to unravel the complexities of human genetics, integrating such advancements into clinical practice will be key to realizing the full potential of personalized medicine. The success of this endeavor will depend on our ability to adapt and refine our methodologies in response to the rapid pace of genomic discoveries, ensuring that pharmacogenomics remains a vital tool in the pursuit of more effective and safer therapeutic strategies.

## Figures and Tables

**Table 1:** The number of constructed haplogroups, observed star alleles in the 1KGP, and total star alleles from CPIC guidelines. The gene length was annotated by Ensembl. Assessment of similarity between haplogroups and star alleles. The similarity between haplogroups and star alleles was computed using the variation of information (VI) index.

Gene	Gene Length ^a^	Haplogroups (Variant)	Observed Star Alleles ^b^ (Variant)	TotalStar Alleles ^c^ (Variant)	VI
IFNL3	1.40	8 (4)	2 (1)	4 (3)	0.98
GSTP1	3.06	4 (2)	3 (2)	4 (2)	1.37
CYP2D6	4.42	13 (4)	38 (37)	131 (125)	1.76
VKORC1	5.14	7 (5)	2 (1)	2 (1)	1.04
NUDT15	9.66	2 (1)	7 (5)	20 (18)	1.23
NAT2	9.97	8 (3)	11 (10)	18 (17)	0.96
UGT1A1	13.05	4 (2)	5 (3)	9 (6)	1.36
G6PD	16.18	2 (1)	21 (19)	186 (182)	1.38
CYP4F2	20.10	4 (2)	3 (2)	4 (2)	2.00
GSTM1	21.23	16 (4)	2 (1)	3 (1)	3.24
UGT2B15	24.00	12 (4)	5 (3)	11 (4)	1.35
TPMT	26.76	4 (2)	13 (12)	46 (45)	1.47
CYP2B6	27.10	13 (4)	15 (7)	37 (35)	2.10
CYP3A4	27.29	8 (3)	20 (19)	33 (32)	2.28
CYP3A5	31.81	5 (3)	4 (3)	9 (8)	0.88
CYP2C8	32.73	7 (3)	11 (11)	18 (18)	2.40
CYP2C9	50.73	4 (2)	21 (20)	71 (69)	1.57
NAT1	53.21	14 (5)	7 (6)	11 (10)	2.27
UGT1A4	54.52	4 (2)	7 (10)	12 (12)	1.98
CACNA1S	73.05	8 (3)	1 (0)	3 (2)	2.37
SLCO1B1	108.05	16 (4)	23 (16)	44 (31)	3.06
RYR1	153.87	8 (3)	2 (1)	49 (48)	1.27
CYP2C19	165.11	8 (3)	17 (17)	36 (33)	2.15
CFTR	250.19	9 (4)	9 (8)	41 (40)	2.05
DPYD	843.31	4 (2)	39 (39)	83 (83)	4.08

^a^ Total gene length by Ensembl in kilo base pairs. ^b^ Total number of observed star alleles in the 1000 Genomes Project of CPIC guidelines, as of 21 October 2021. ^c^ Total number of star alleles of CPIC guidelines, as of 21 October 2021.

## Data Availability

The 1000 Genomes Project data can be accessed at https://www.internationalgenome.org/data/ (accessed on 2 April 2024).

## Supplementary Materials

The following supporting information can be downloaded at: https://www.mdpi.com/article/10.3390/genes15040521/s1, Figure S1: The cumulative density of the number of star allele defining variants; Figure S2: Workflow of the schema; Figure S3: The correlation coefficient among gene length, the minor allele frequency at the stopping condition, and the variation of information; Figures S4–S28: The network plot of haplogroups and star alleles for each of the 25 pharmacogenes; Figure S29: The distribution of the number of variants defining haplogroup-independent star alleles; Table S1: The star-allele nomenclature.
