# Supplementary material for: Contradiction in Star-Allele Nomenclature of Pharmacogenes between Common Haplotypes and Rare Variants
Source: Genes (Basel). 2024 Apr 22;15(4):521. doi: 10.3390/genes15040521 (PMC11050392; doi:10.3390/genes15040521)
Supplement: Supplementary file 1 [file genes-15-00521-s001.zip › Supplementary_Figures.pdf]

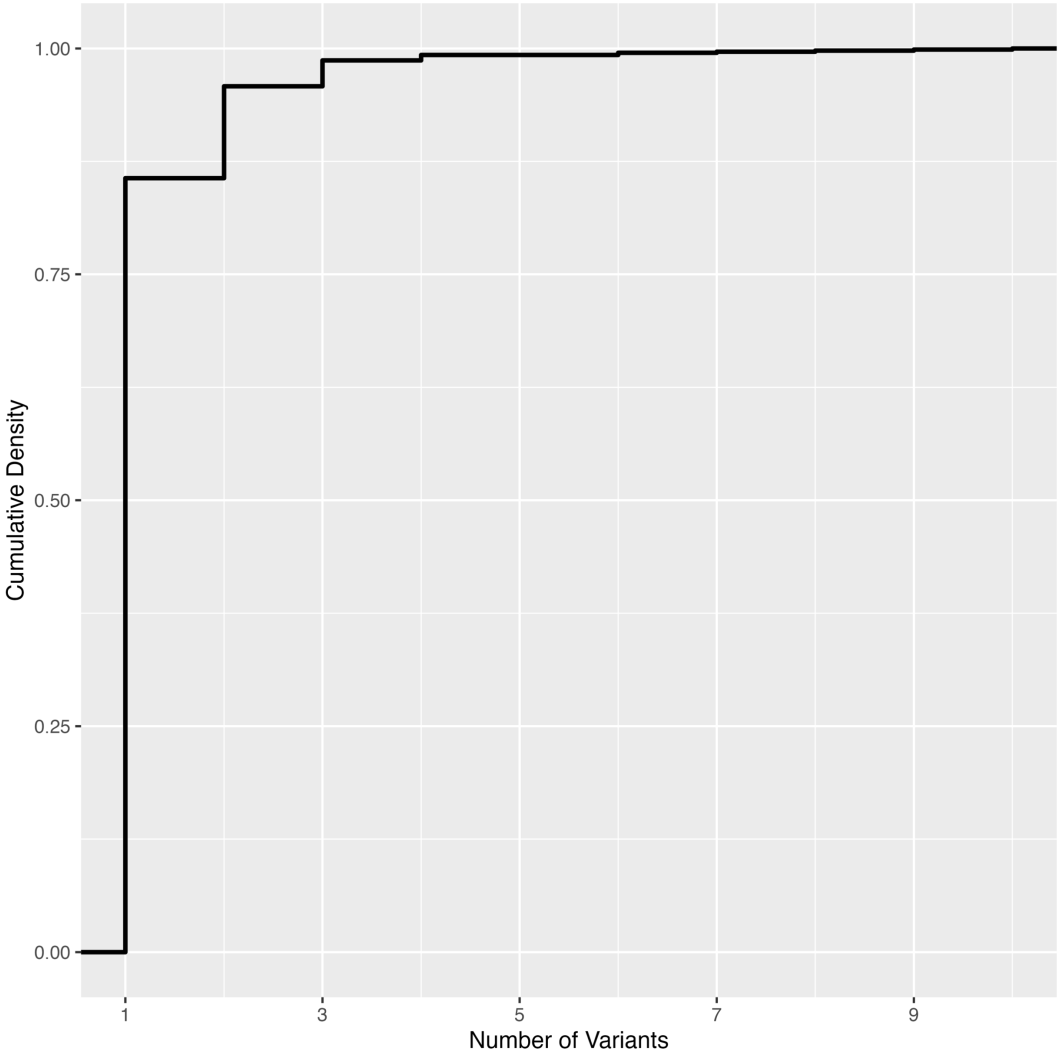

Supplementary Figure S1. The number of variants defining star alleles for 25 pharmacogenes are represented as the cumulative density plot.

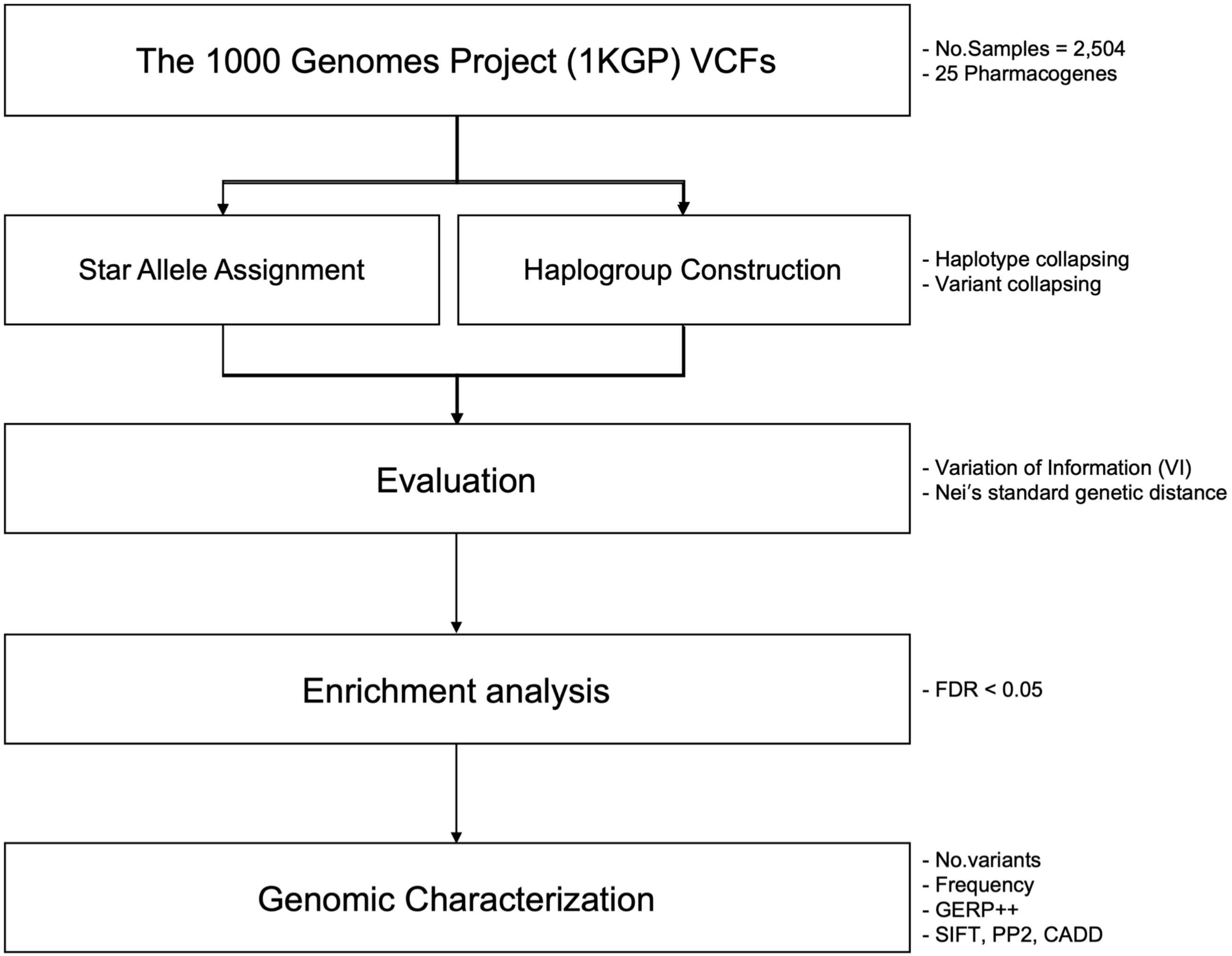

Supplementary Figure S2. Workflow schema of study.

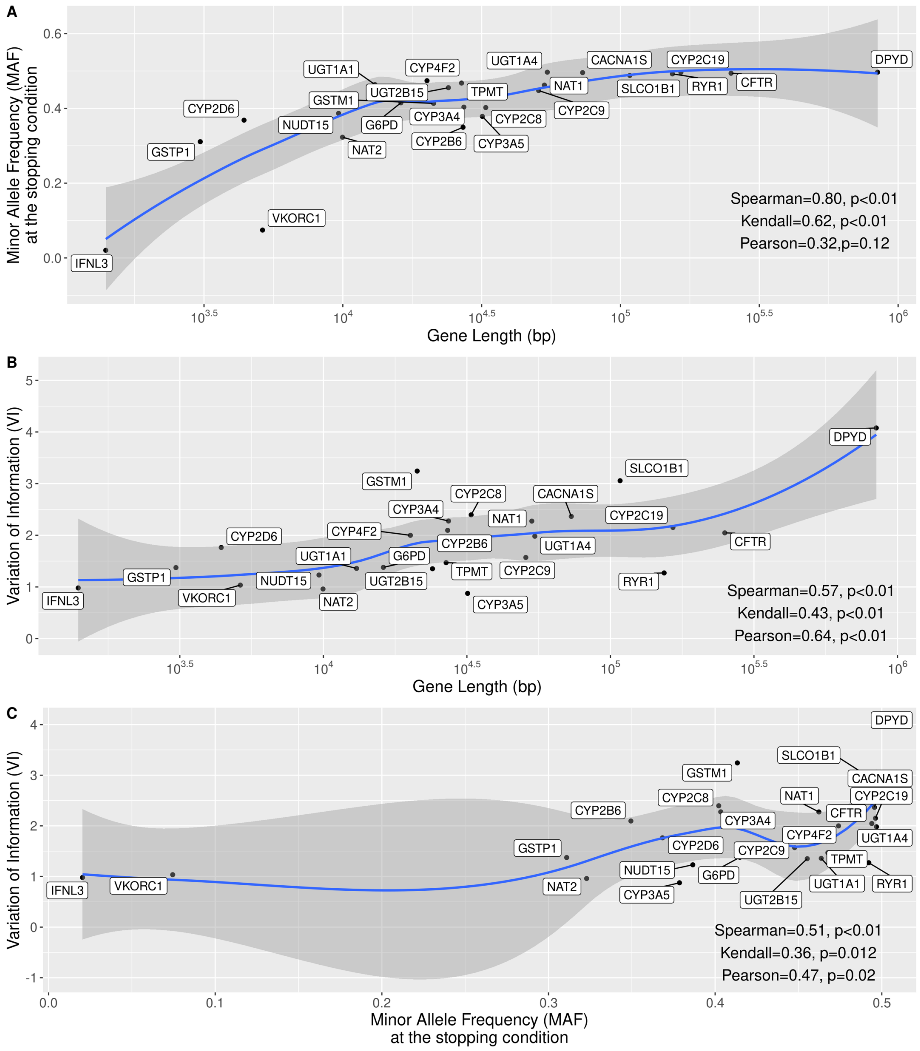

Supplementary Figure S3. The distribution between (A) gene length and minor allele frequency (MAF), (B) gene length and the variation of information (VI), and (C) MAF and VI. The correlation coefficients of Spearman, Kendall, and Pearson are shown.

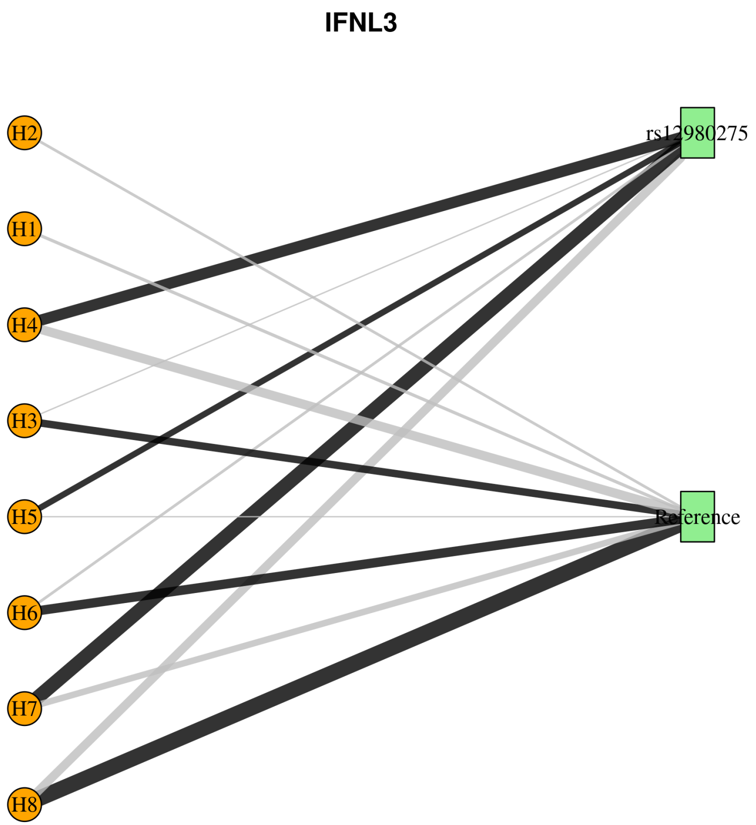

Supplementary Figure S4. A network plot of IFNL3. The orange circular nodes represent haplogroups, and the green rectangular nodes denote star alleles. Black edges indicate significant associations at FDR < 0.05. The width of each edge reflects to the frequency of the association.

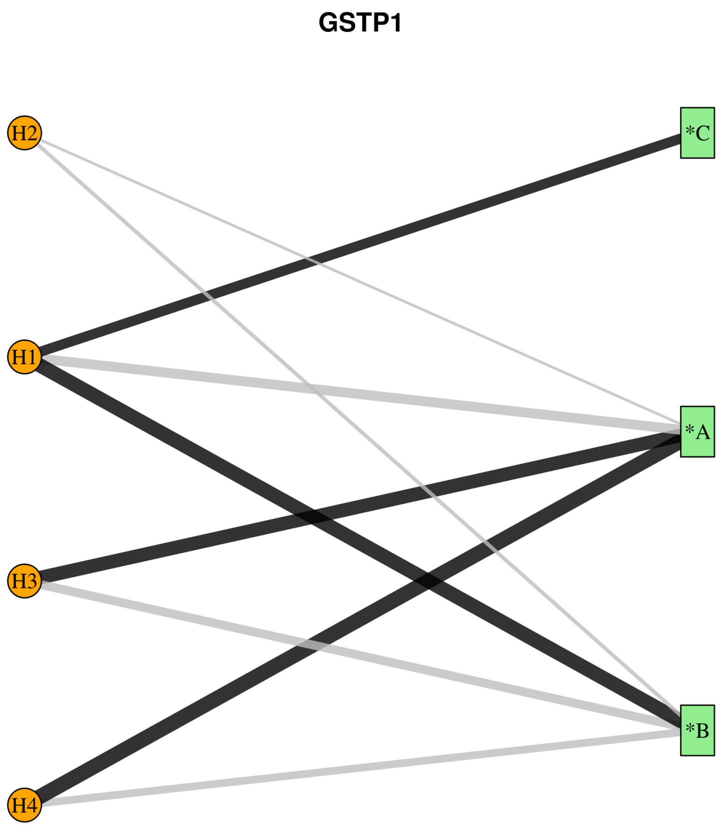

Supplementary Figure S5. A network plot of GSTP1. The orange circular nodes represent haplogroups, and the green rectangular nodes denote star alleles. Black edges indicate significant associations at FDR < 0.05. The width of each edge reflects to the frequency of the association.

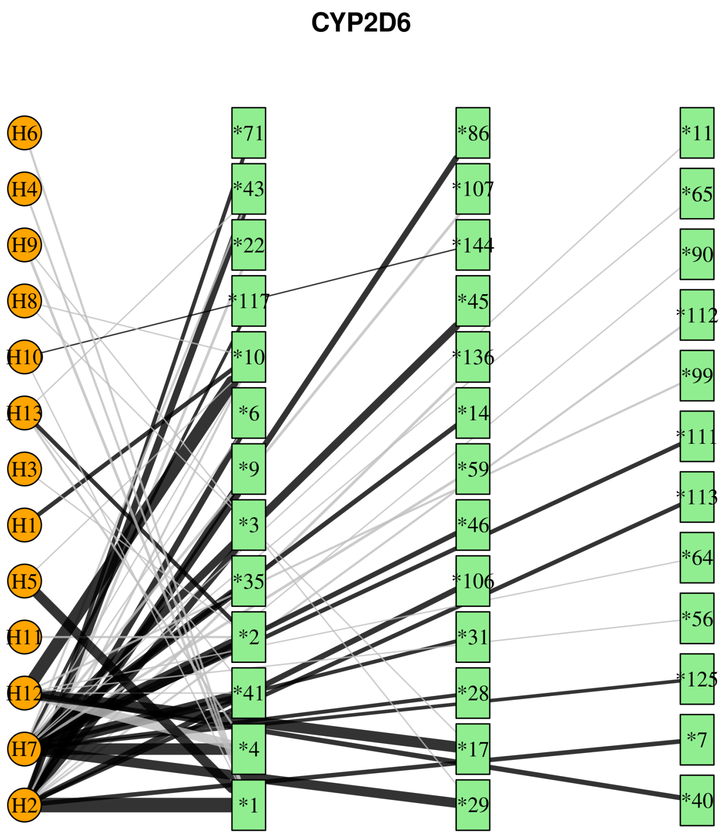

Supplementary Figure S6. A network plot of CYP2D6. The orange circular nodes represent haplogroups, and the green rectangular nodes denote star alleles. Black edges indicate significant associations at FDR < 0.05. The width of each edge reflects to the frequency of the association.

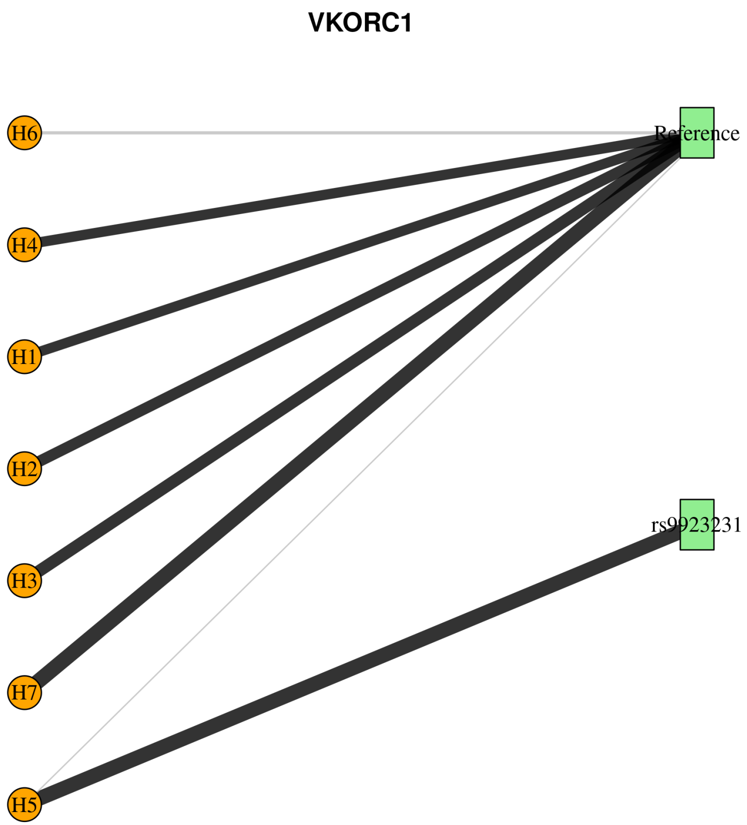

Supplementary Figure S7. A network plot of VKORC1. The orange circular nodes represent haplogroups, and the green rectangular nodes denote star alleles. Black edges indicate significant associations at FDR < 0.05. The width of each edge reflects to the frequency of the association.

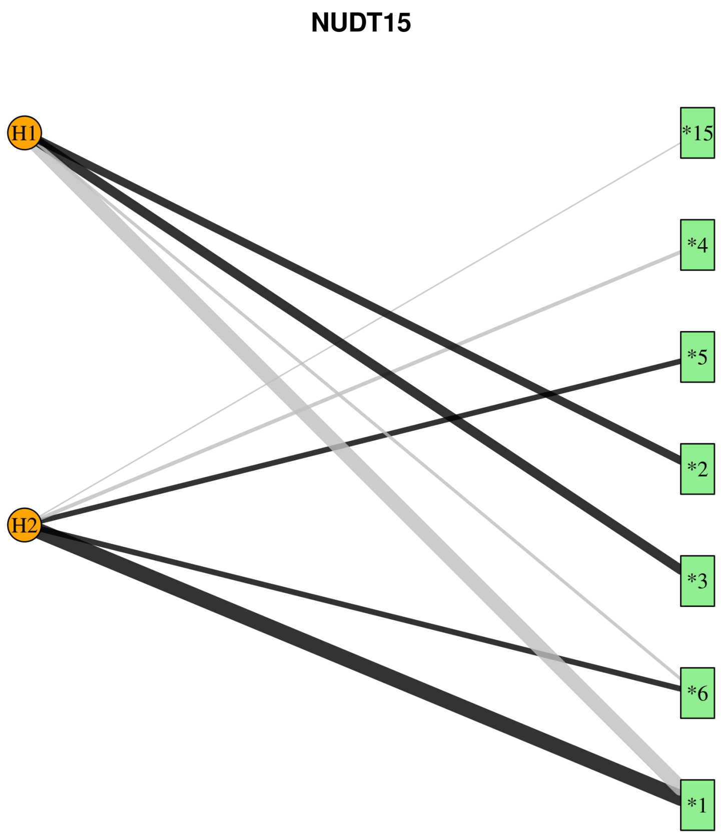

Supplementary Figure S8. A network plot of NUDT15. The orange circular nodes represent haplogroups, and the green rectangular nodes denote star alleles. Black edges indicate significant associations at FDR < 0.05. The width of each edge reflects to the frequency of the association.

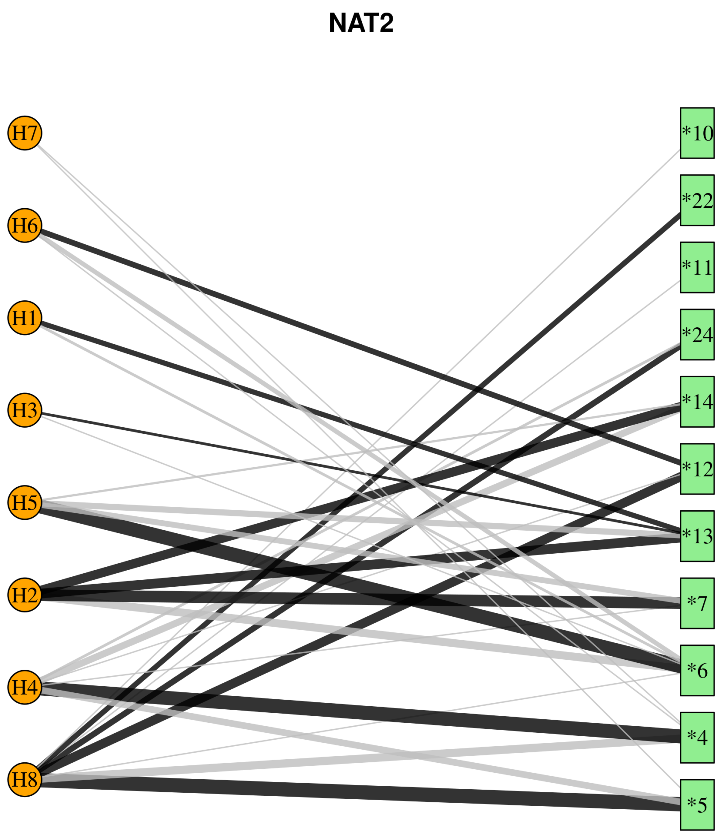

Supplementary Figure S9. A network plot of NAT2. The orange circular nodes represent haplogroups, and the green rectangular nodes denote star alleles. Black edges indicate significant associations at FDR < 0.05. The width of each edge reflects to the frequency of the association.

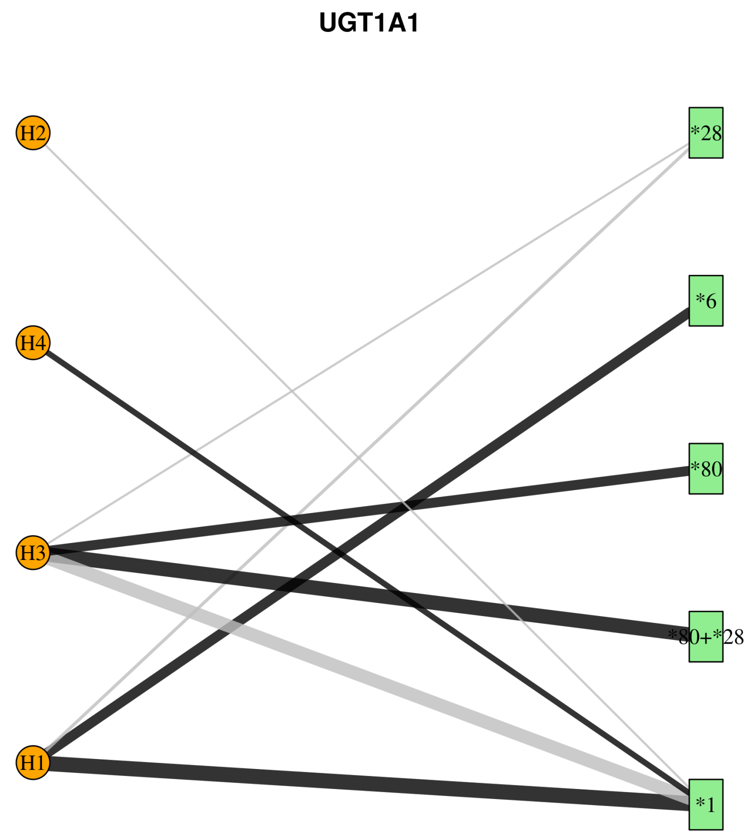

Supplementary Figure S10. A network plot of UGT1A1. The orange circular nodes represent haplogroups, and the green rectangular nodes denote star alleles. Black edges indicate significant associations at FDR < 0.05. The width of each edge reflects to the frequency of the association.

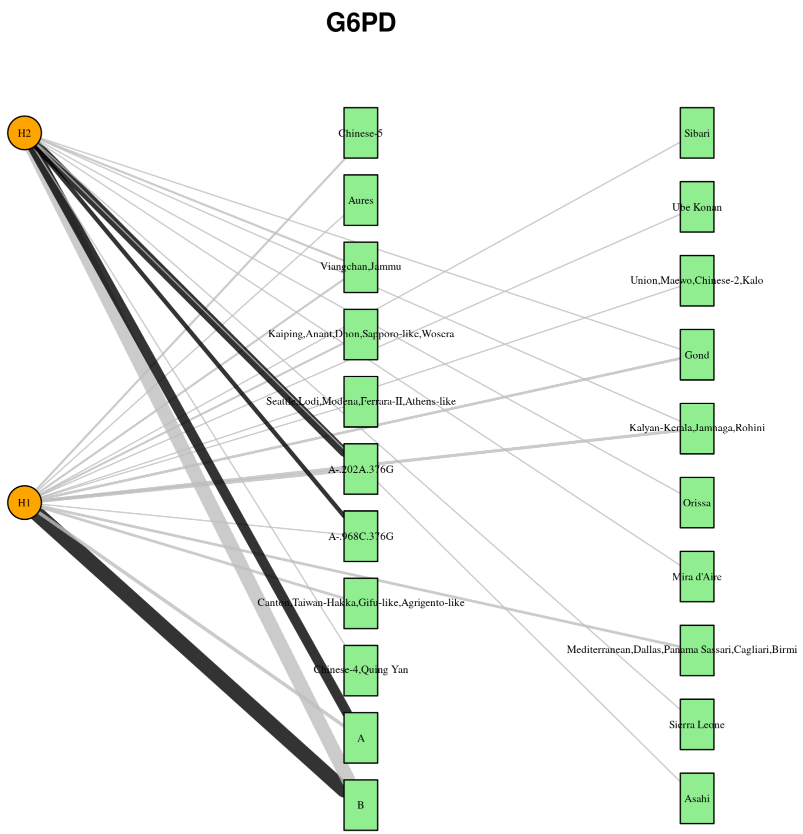

Supplementary Figure S11. A network plot of G6PD. The orange circular nodes represent haplogroups, and the green rectangular nodes denote star alleles. Black edges indicate significant associations at FDR < 0.05. The width of each edge reflects to the frequency of the association.

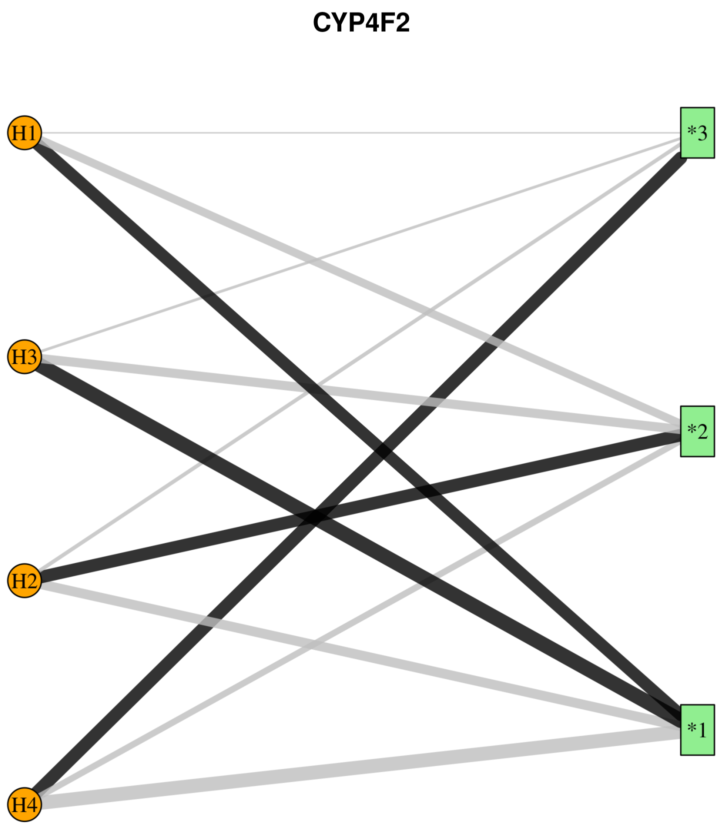

Supplementary Figure S12. A network plot of CYP4F2. The orange circular nodes represent haplogroups, and the green rectangular nodes denote star alleles. Black edges indicate significant associations at FDR < 0.05. The width of each edge reflects to the frequency of the association.

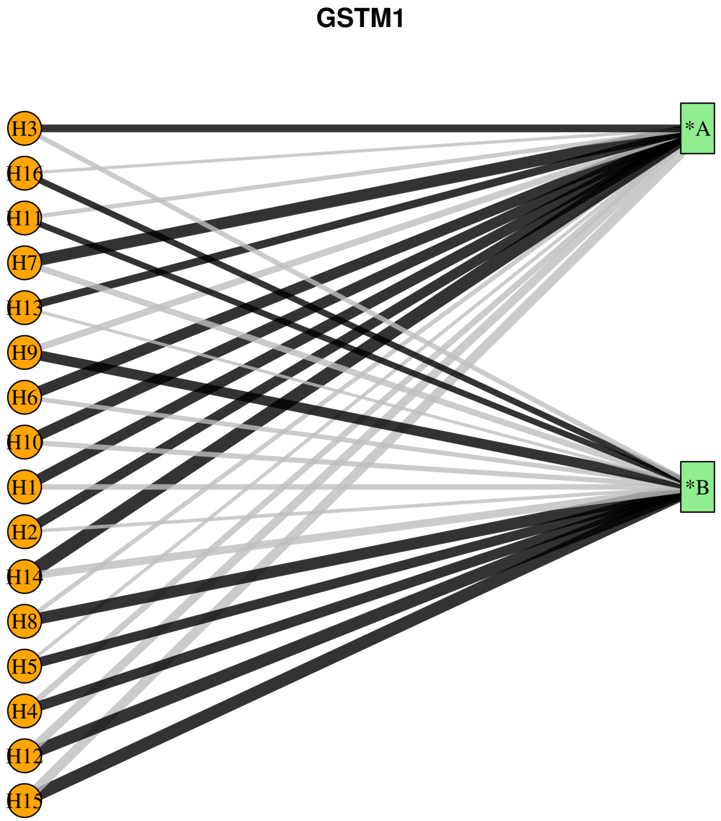

Supplementary Figure S13. A network plot of GSTM1. The orange circular nodes represent haplogroups, and the green rectangular nodes denote star alleles. Black edges indicate significant associations at FDR < 0.05. The width of each edge reflects to the frequency of the association.

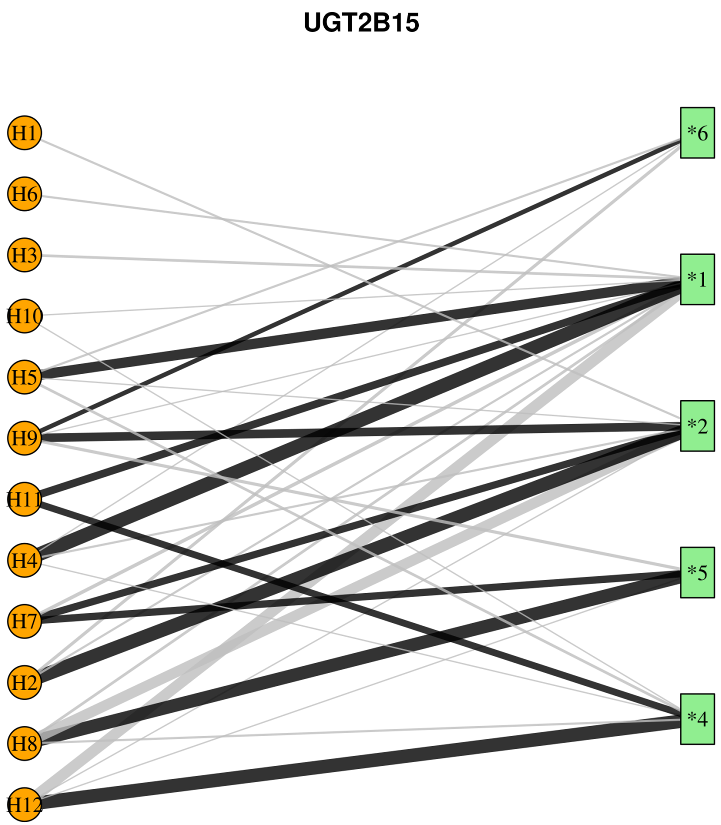

Supplementary Figure S14. A network plot of UGT2B15. The orange circular nodes represent haplogroups, and the green rectangular nodes denote star alleles. Black edges indicate significant associations at FDR < 0.05. The width of each edge reflects to the frequency of the association.

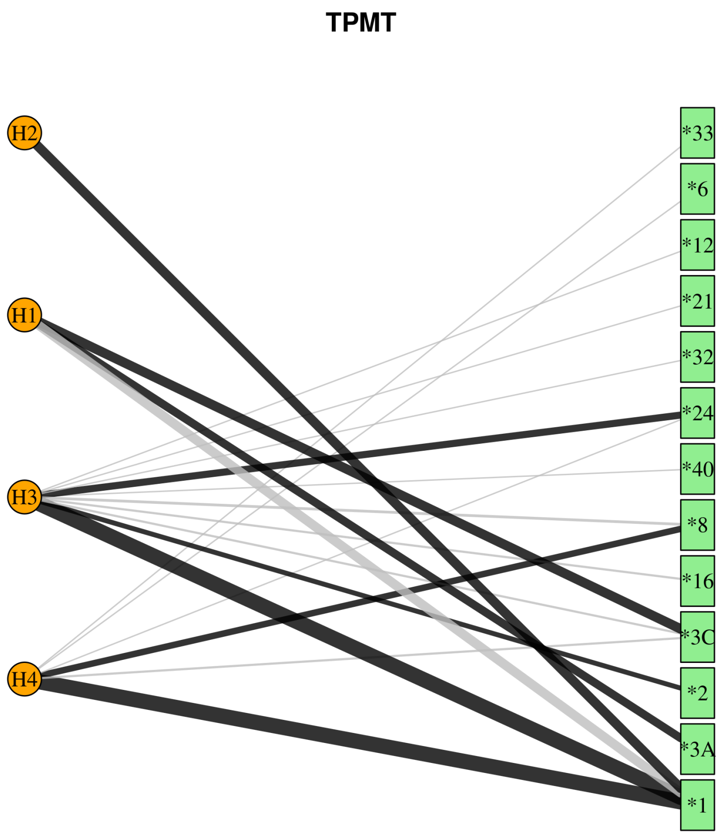

Supplementary Figure S15. A network plot of TPMT. The orange circular nodes represent haplogroups, and the green rectangular nodes denote star alleles. Black edges indicate significant associations at FDR < 0.05. The width of each edge reflects to the frequency of the association.

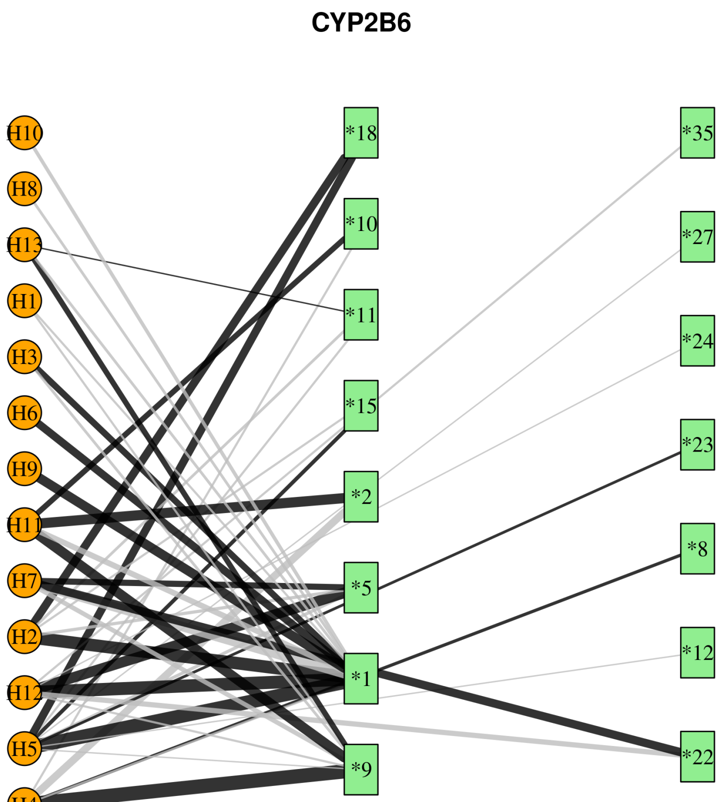

Supplementary Figure S16. A network plot of CYP2B6. The orange circular nodes represent haplogroups, and the green rectangular nodes denote star alleles. Black edges indicate significant associations at FDR < 0.05. The width of each edge reflects to the frequency of the association.

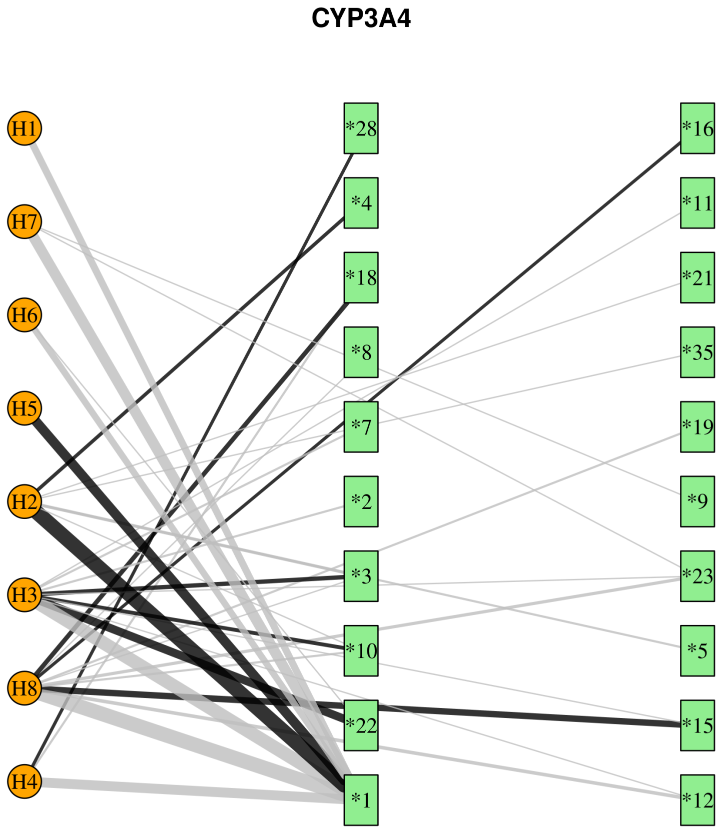

Supplementary Figure S17. A network plot of CYP3A4. The orange circular nodes represent haplogroups, and the green rectangular nodes denote star alleles. Black edges indicate significant associations at FDR < 0.05. The width of each edge reflects to the frequency of the association.

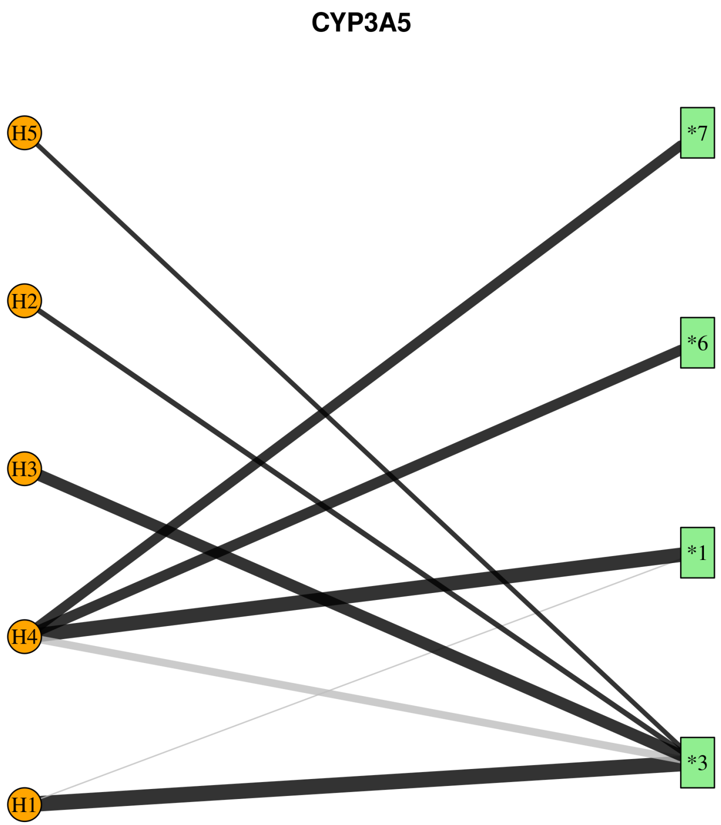

Supplementary Figure S18. A network plot of CYP3A5. The orange circular nodes represent haplogroups, and the green rectangular nodes denote star alleles. Black edges indicate significant associations at FDR < 0.05. The width of each edge reflects to the frequency of the association.

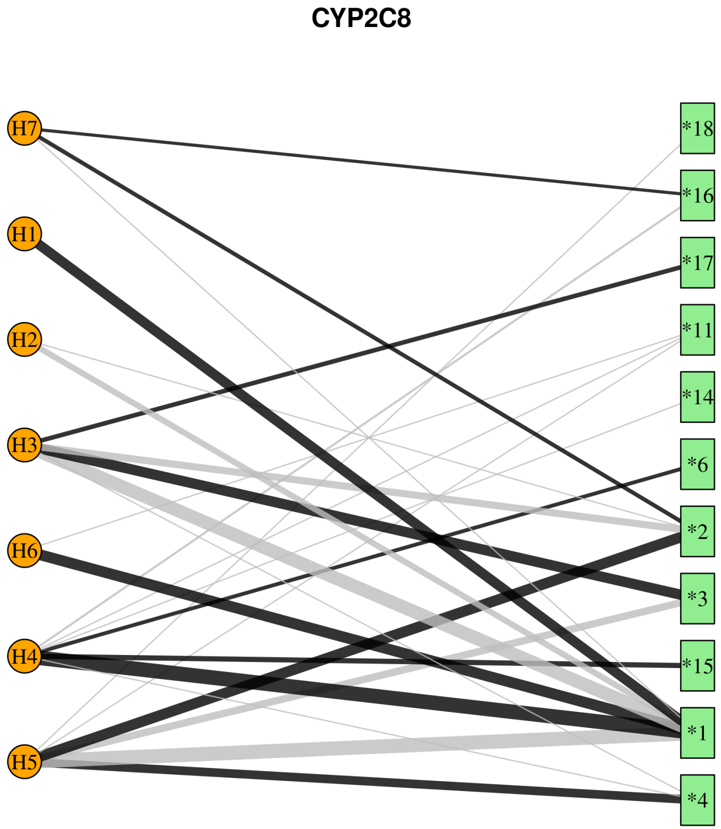

Supplementary Figure S19. A network plot of CYP2C8. The orange circular nodes represent haplogroups, and the green rectangular nodes denote star alleles. Black edges indicate significant associations at FDR < 0.05. The width of each edge reflects to the frequency of the association.

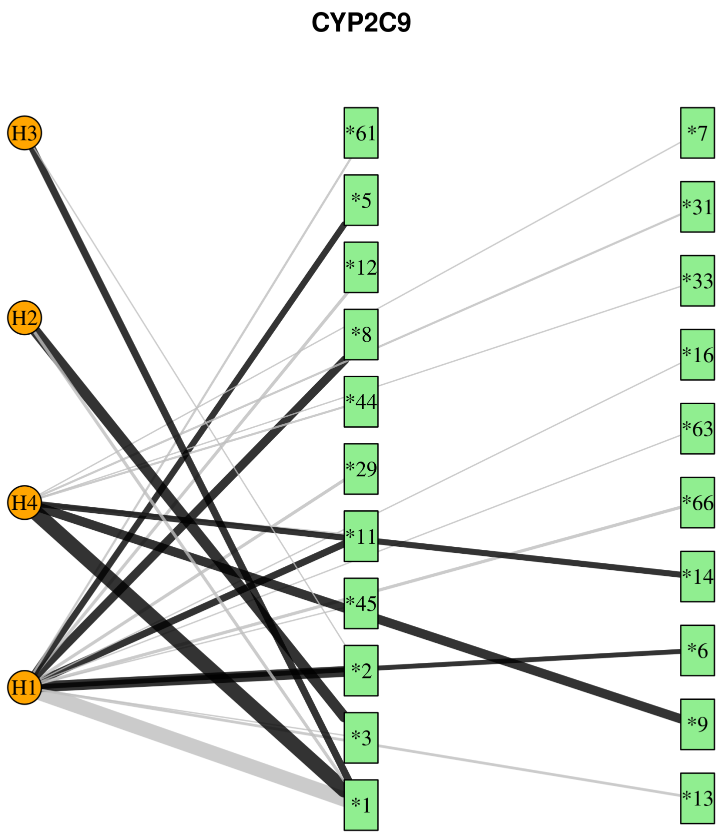

Supplementary Figure S20. A network plot of CYP2C9. The orange circular nodes represent haplogroups, and the green rectangular nodes denote star alleles. Black edges indicate significant associations at FDR < 0.05. The width of each edge reflects to the frequency of the association.

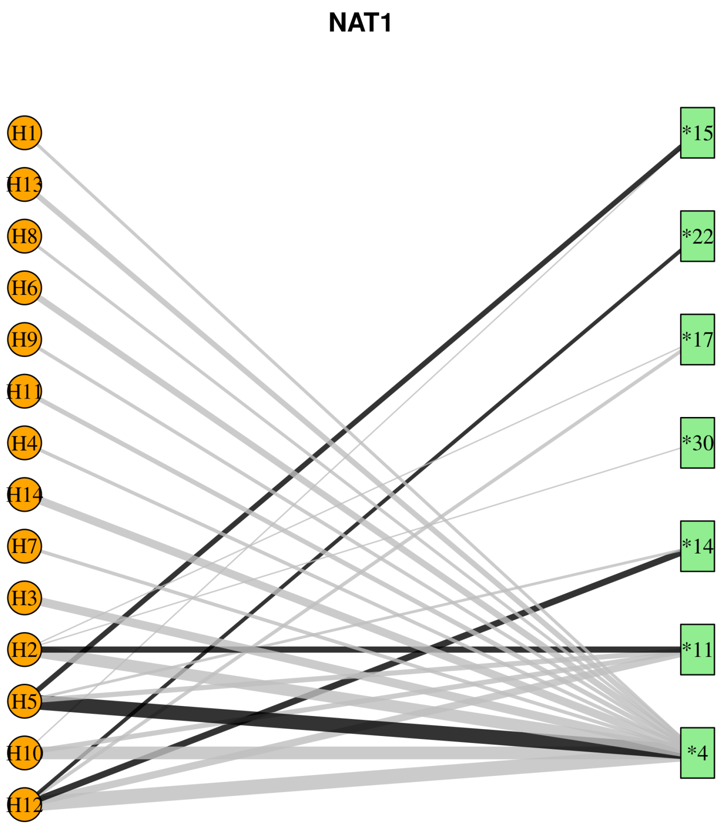

Supplementary Figure S21. A network plot of NAT1. The orange circular nodes represent haplogroups, and the green rectangular nodes denote star alleles. Black edges indicate significant associations at FDR < 0.05. The width of each edge reflects to the frequency of the association.

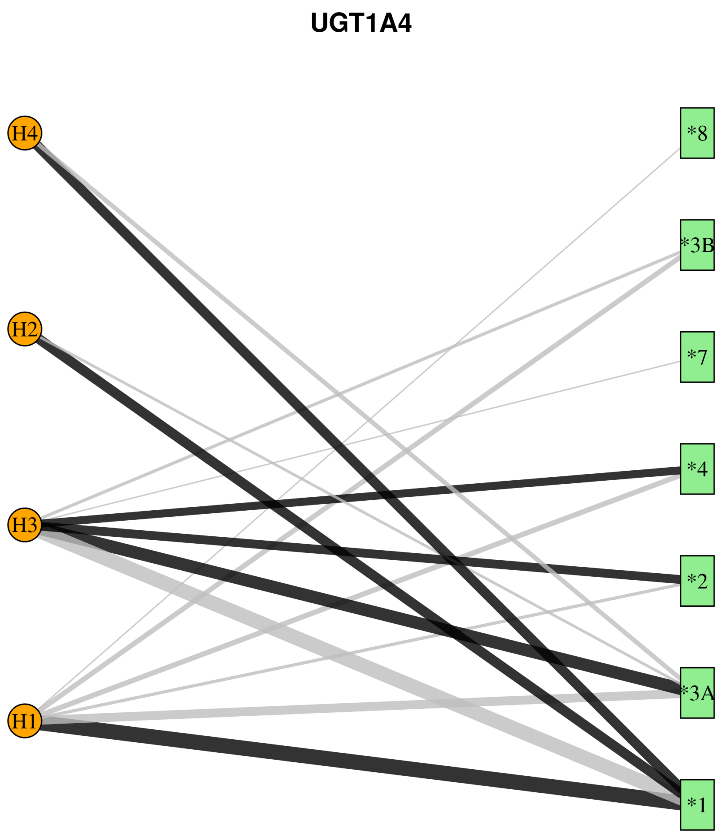

Supplementary Figure S22. A network plot of UGT1A4. The orange circular nodes represent haplogroups, and the green rectangular nodes denote star alleles. Black edges indicate significant associations at FDR < 0.05. The width of each edge reflects to the frequency of the association.

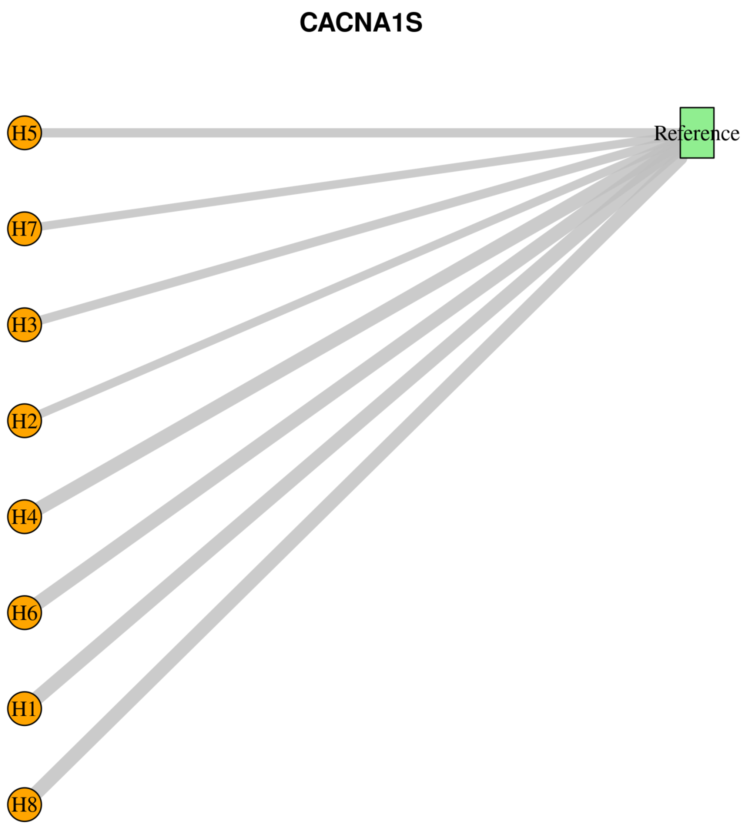

Supplementary Figure S23. A network plot of CACNA1S. The orange circular nodes represent haplogroups, and the green rectangular nodes denote star alleles. Black edges indicate significant associations at FDR < 0.05. The width of each edge reflects to the frequency of the association.

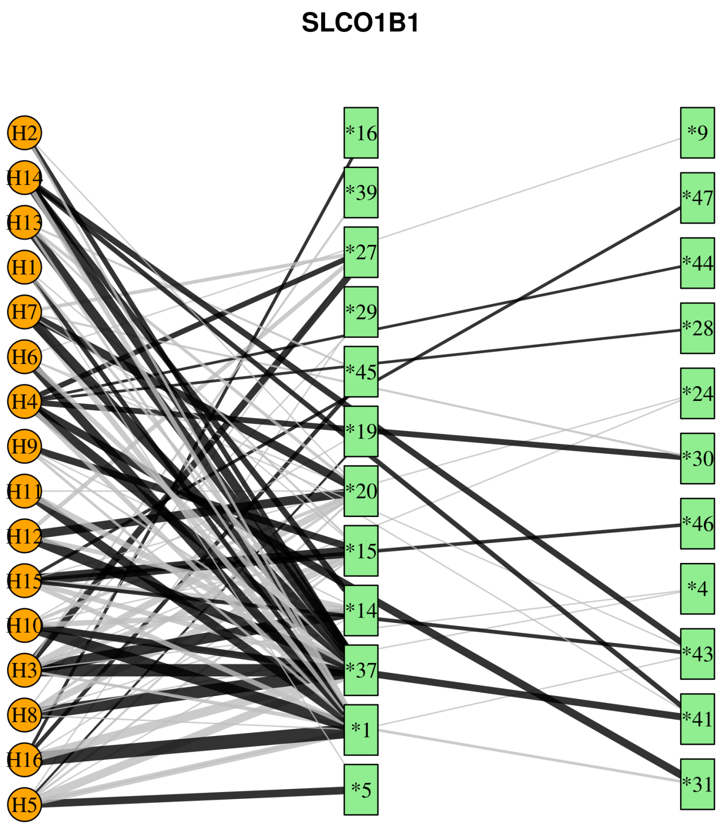

Supplementary Figure S24. A network plot of SLCO1B1. The orange circular nodes represent haplogroups, and the green rectangular nodes denote star alleles. Black edges indicate significant associations at FDR < 0.05. The width of each edge reflects to the frequency of the association.

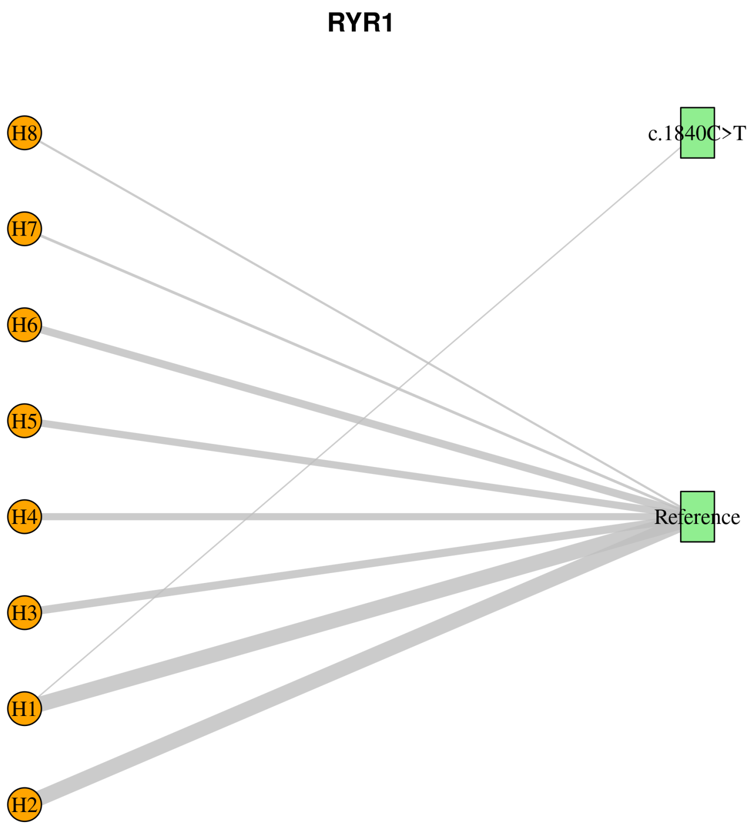

Supplementary Figure S25. A network plot of RYR1. The orange circular nodes represent haplogroups, and the green rectangular nodes denote star alleles. Black edges indicate significant associations at FDR < 0.05. The width of each edge reflects to the frequency of the association.

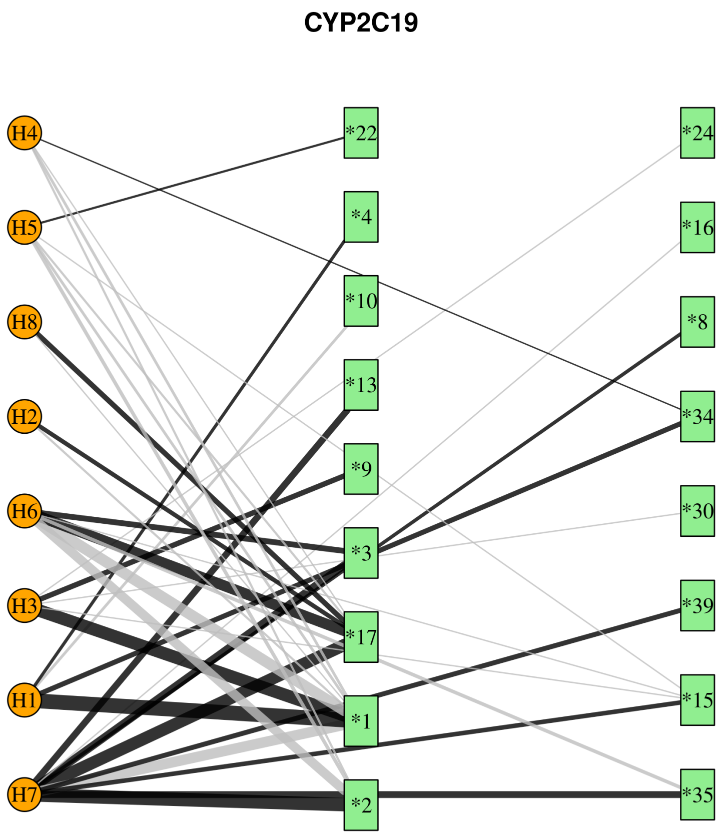

Supplementary Figure S26. A network plot of CYP2C19. The orange circular nodes represent haplogroups, and the green rectangular nodes denote star alleles. Black edges indicate significant associations at FDR < 0.05. The width of each edge reflects to the frequency of the association.

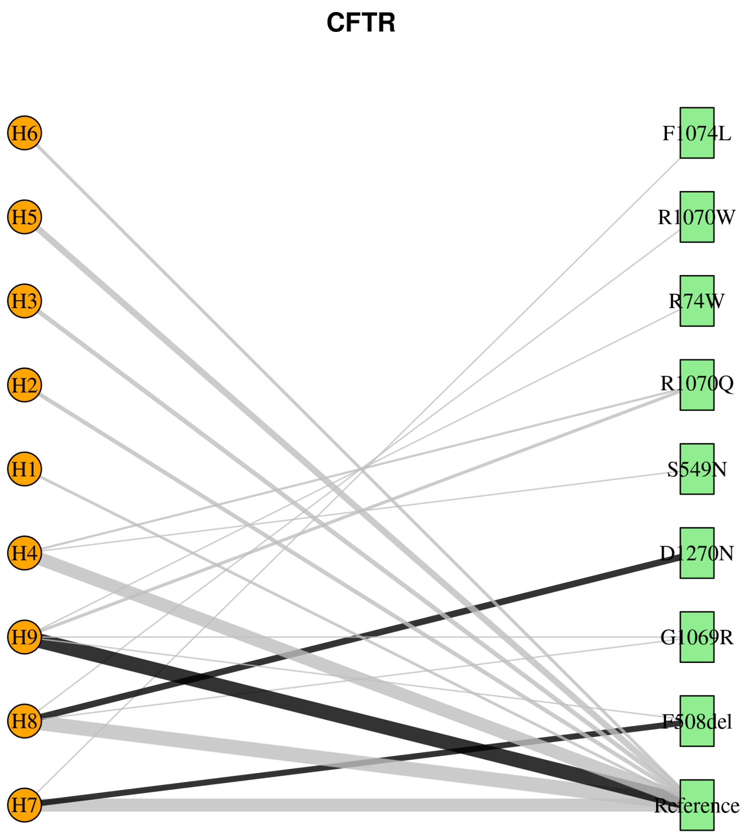

Supplementary Figure S27. A network plot of CFTR. The orange circular nodes represent haplogroups, and the green rectangular nodes denote star alleles. Black edges indicate significant associations at FDR < 0.05. The width of each edge reflects to the frequency of the association.

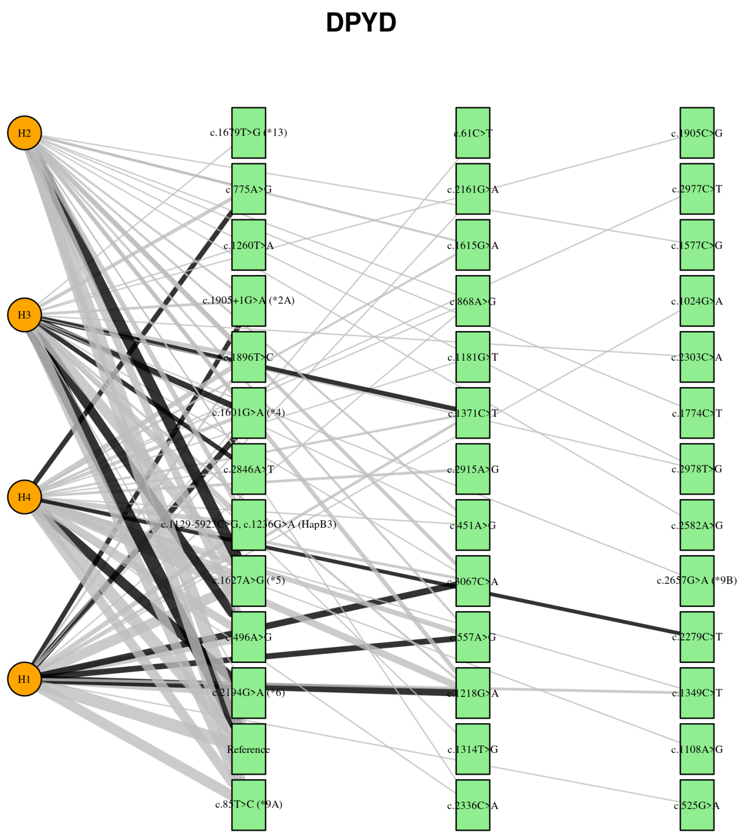

Supplementary Figure S28. A network plot of DPYD. The orange circular nodes represent haplogroups, and the green rectangular nodes denote star alleles. Black edges indicate significant associations at FDR < 0.05. The width of each edge reflects to the frequency of the association.

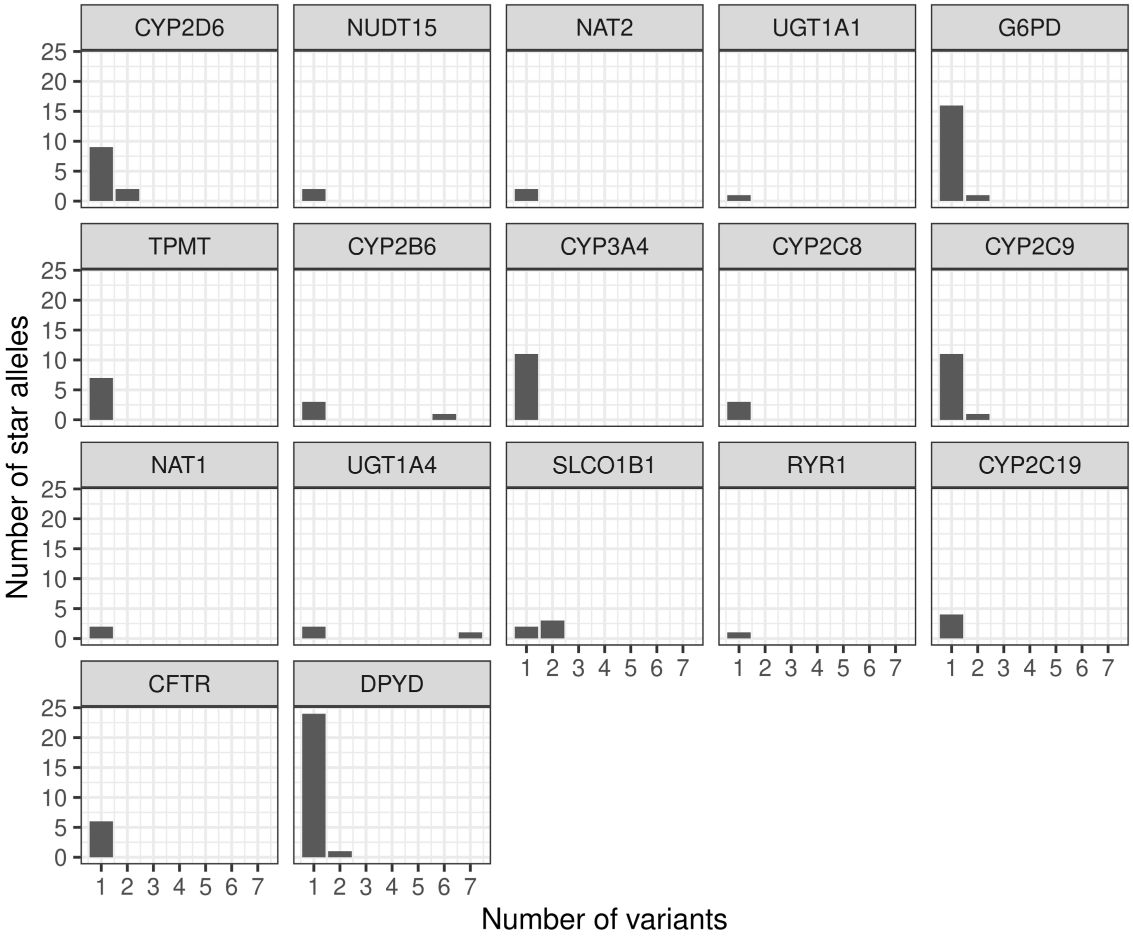

Supplementary Figure S29. The distribution of variant counts that defining S_I alleles. Among 25 pharmacogenes, only 17 genes have at least S_I alleles.
